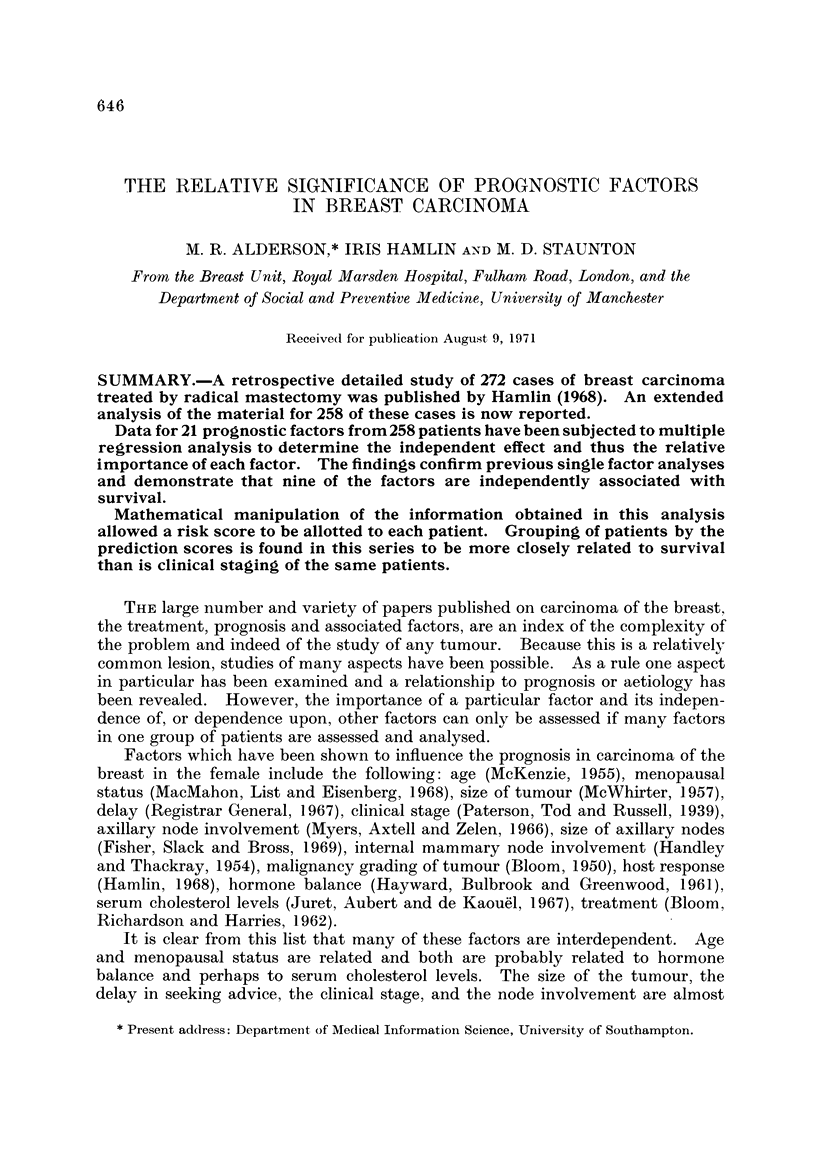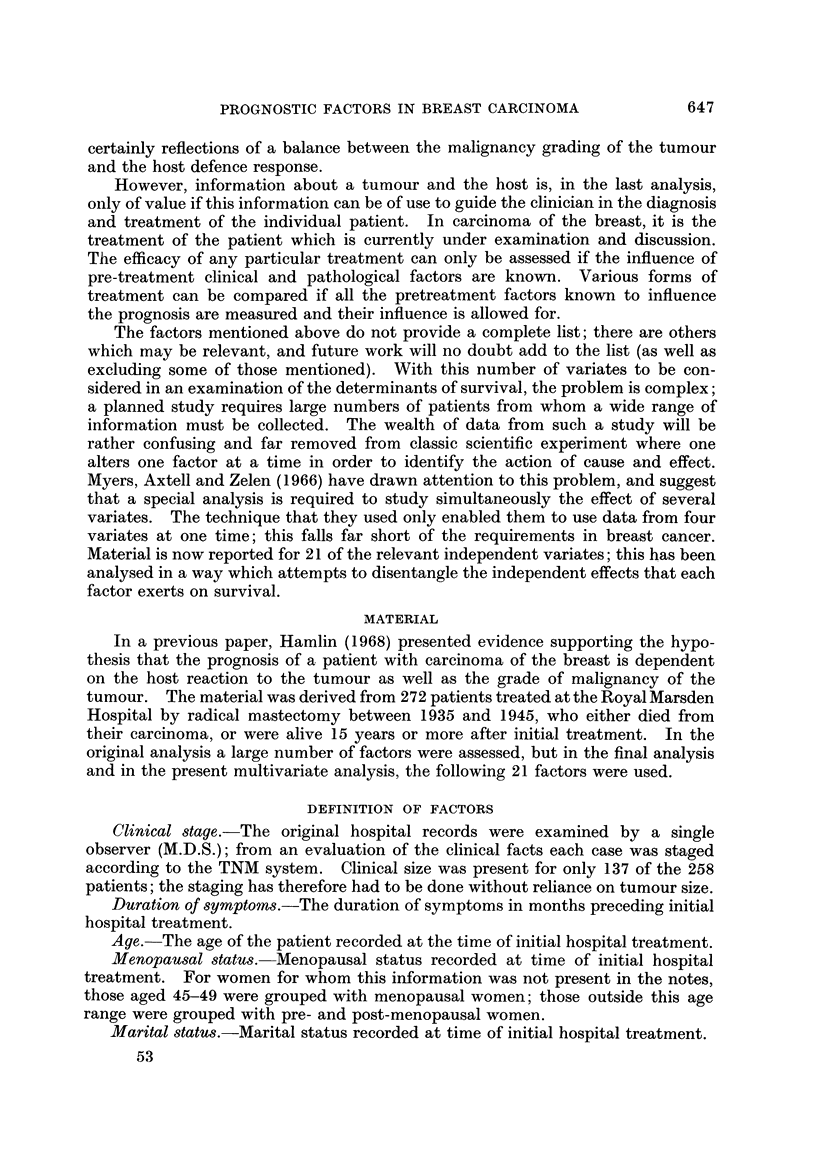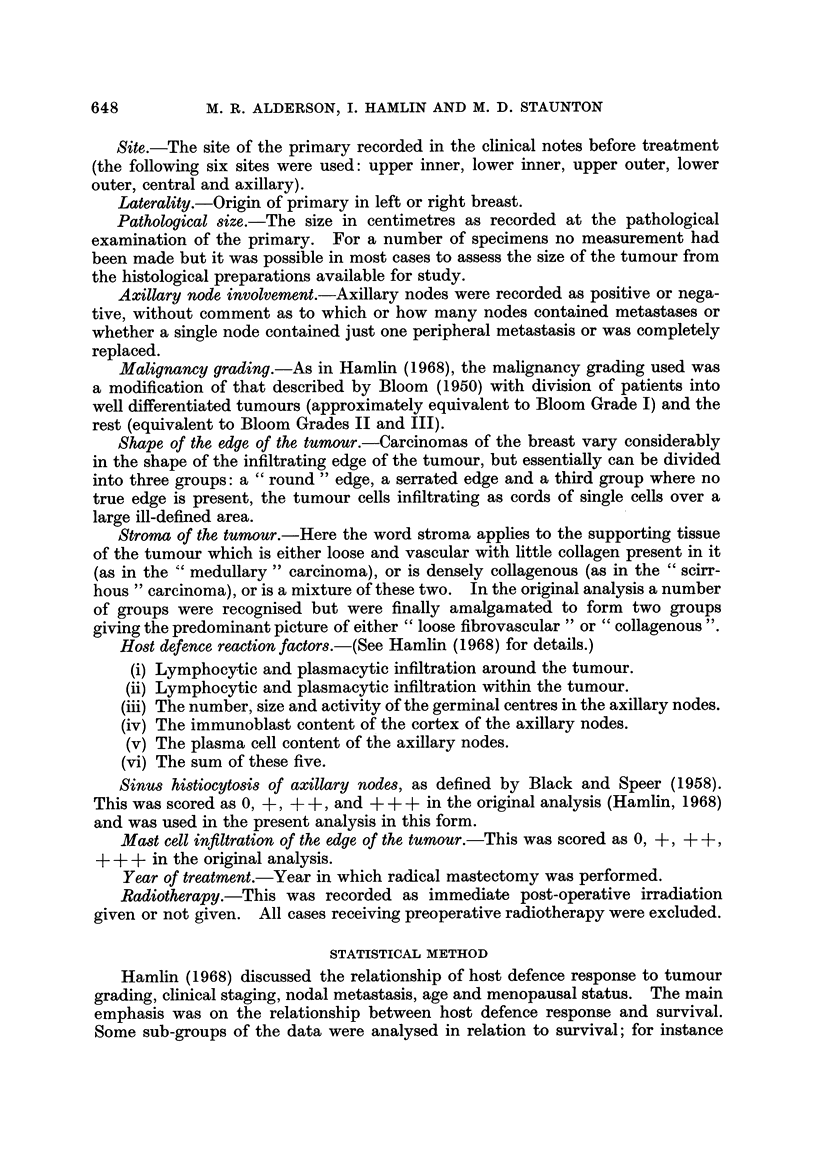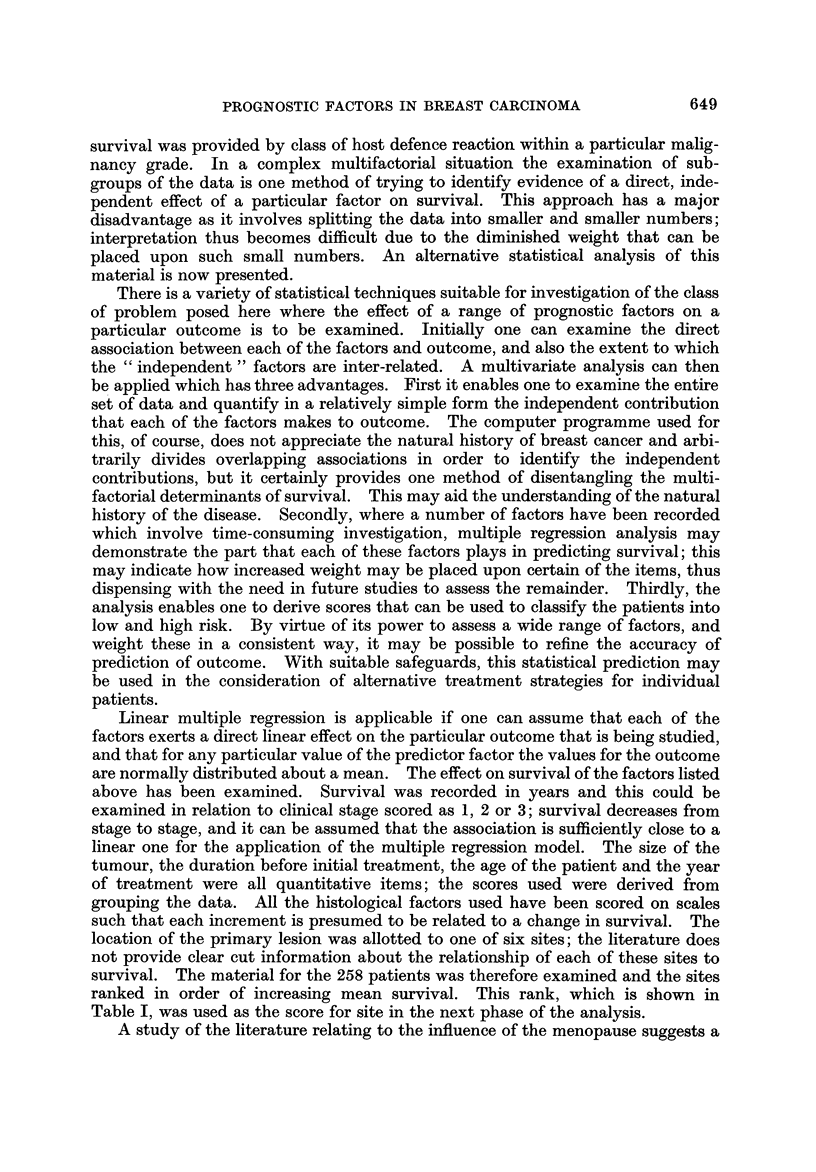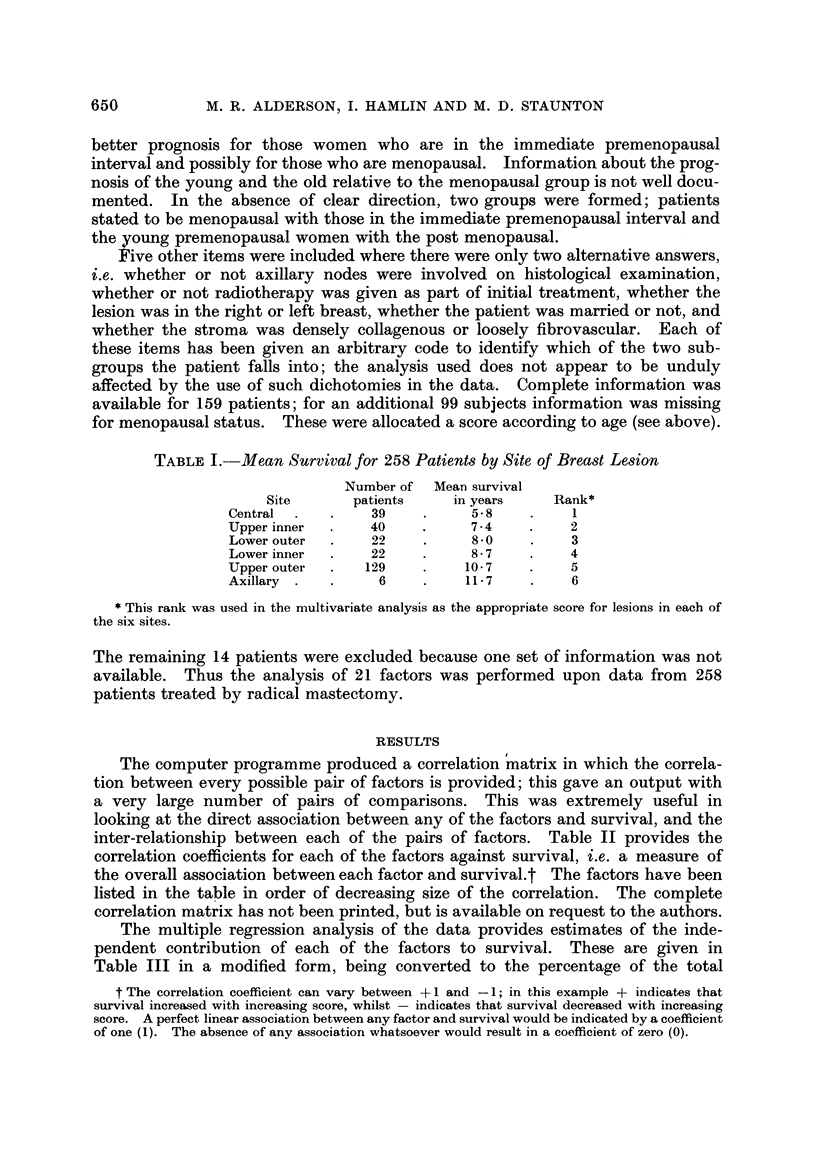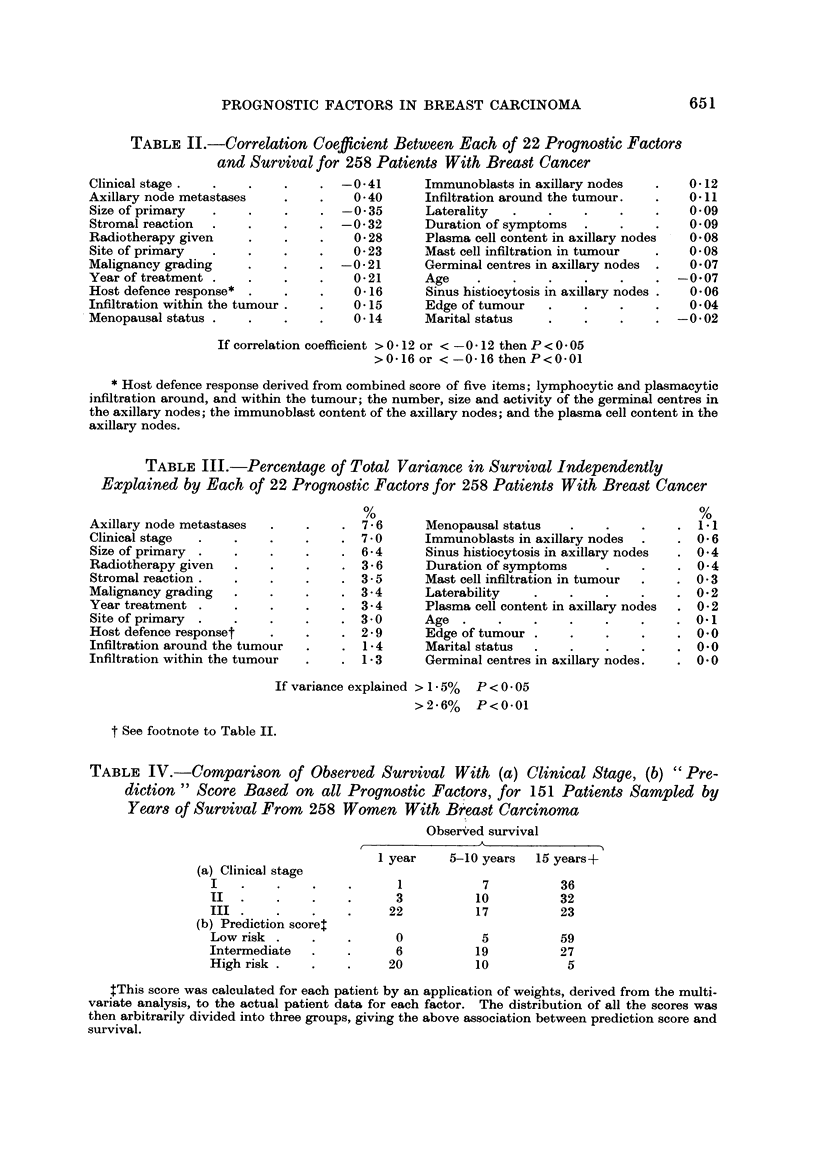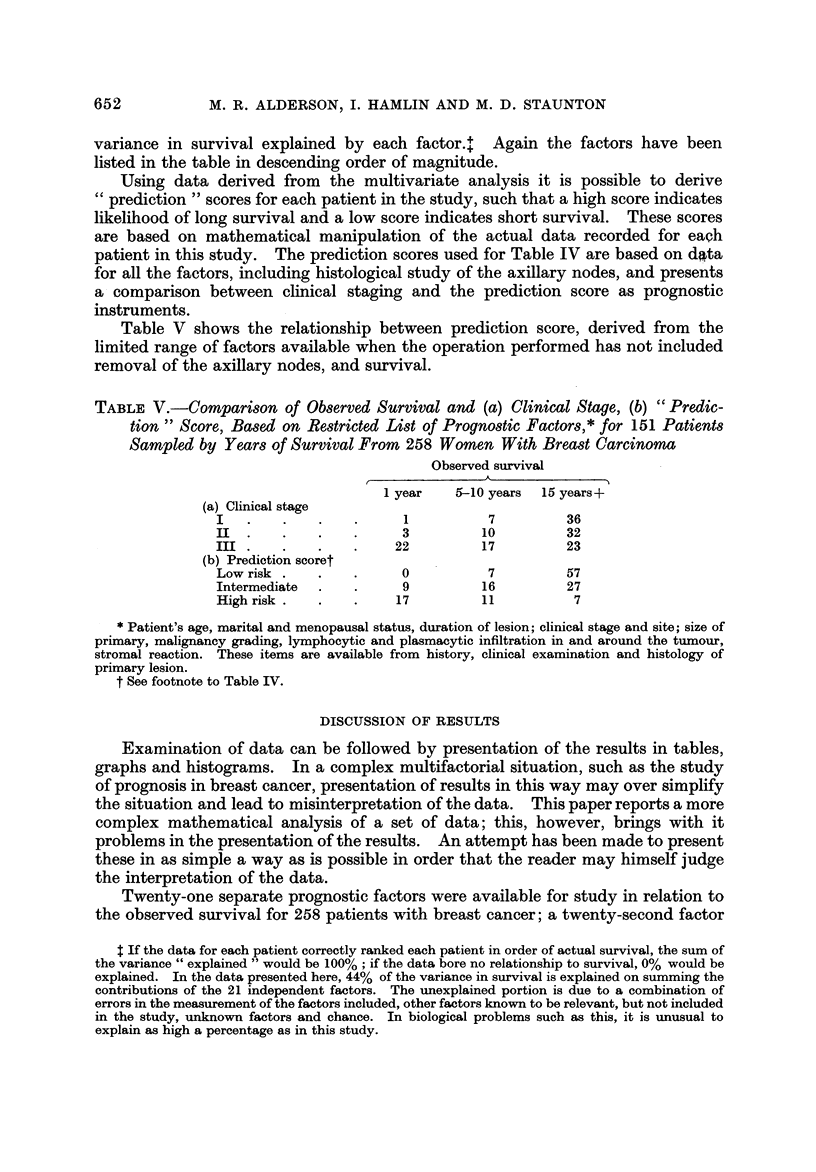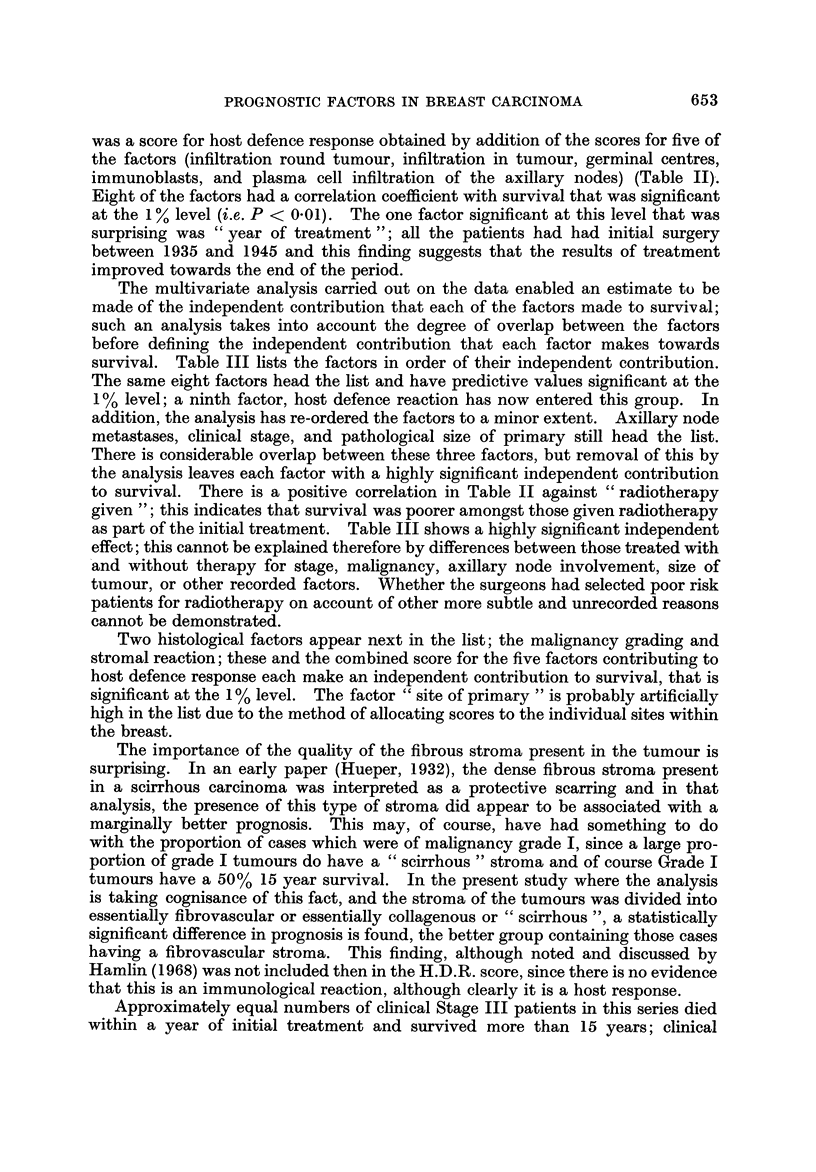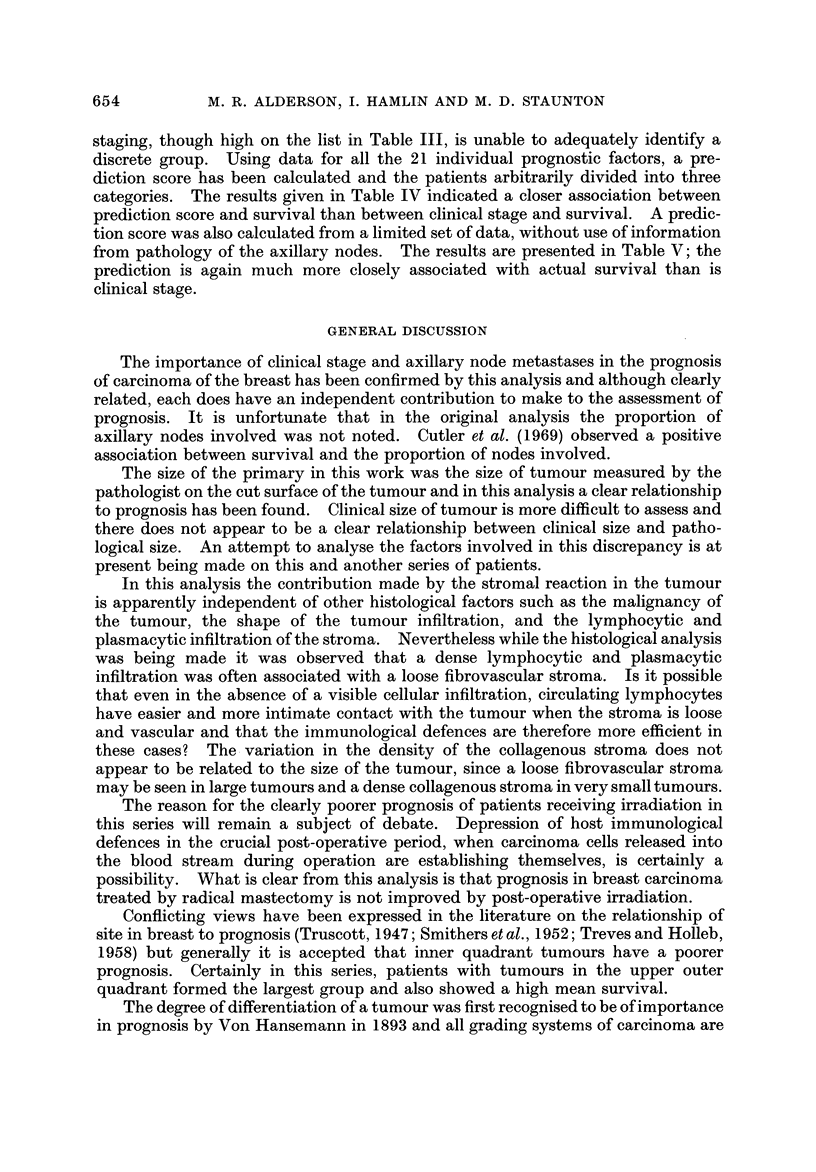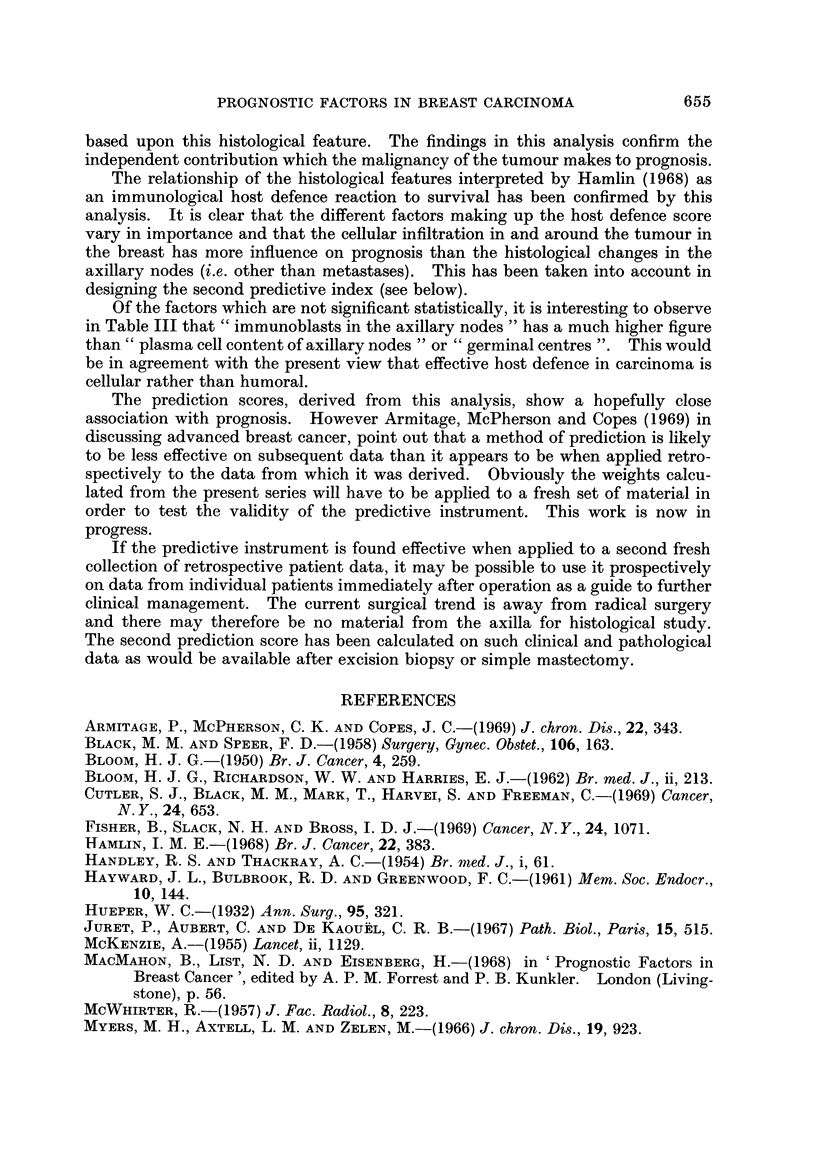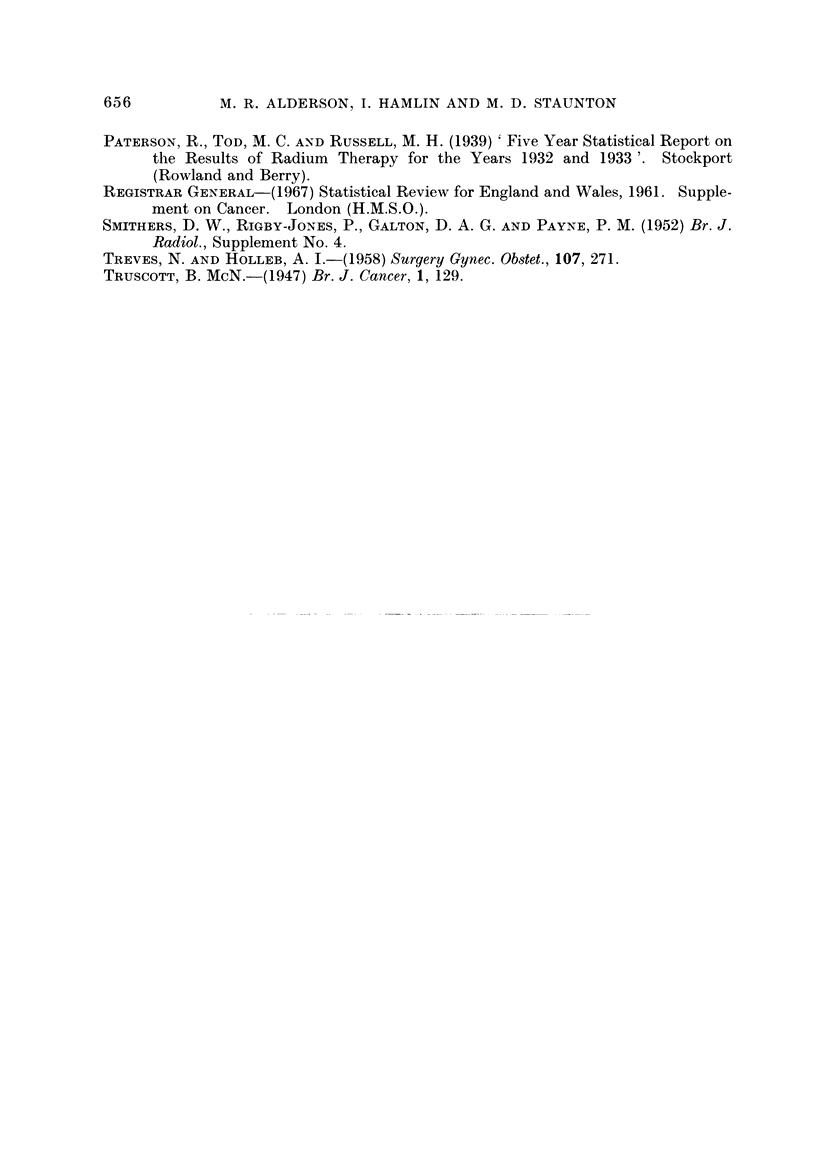# The Relative Significance of Prognostic Factors in Breast Carcinoma

**DOI:** 10.1038/bjc.1971.80

**Published:** 1971-12

**Authors:** M. R. Alderson, Iris Hamlin, M. D. Staunton

## Abstract

A retrospective detailed study of 272 cases of breast carcinoma treated by radical mastectomy was published by Hamlin (1968). An extended analysis of the material for 258 of these cases is now reported.

Data for 21 prognostic factors from 258 patients have been subjected to multiple regression analysis to determine the independent effect and thus the relative importance of each factor. The findings confirm previous single factor analyses and demonstrate that nine of the factors are independently associated with survival.

Mathematical manipulation of the information obtained in this analysis allowed a risk score to be allotted to each patient. Grouping of patients by the prediction scores is found in this series to be more closely related to survival than is clinical staging of the same patients.


### THE RELATIVE SIGNIFICANCE OF PROGNOSTIC FACTORS IN BREAST CARCINOMA

M. R. ALDERSON,* IRIS HAMLIN AND M. D. STAUNTON

*From the Breast Unit, Royal Marsden Hospital, Fulham Road, London, and the Department of Social and Preventive Medicine, University of Manchester*

Received for publication August 9, 1971

**SUMMARY.—A retrospective detailed study of 272 cases of breast carcinoma treated by radical mastectomy was published by Hamlin (1968). An extended analysis of the material for 258 of these cases is now reported.**

**Data for 21 prognostic factors from 258 patients have been subjected to multiple regression analysis to determine the independent effect and thus the relative importance of each factor. The findings confirm previous single factor analyses and demonstrate that nine of the factors are independently associated with survival.**

**Mathematical manipulation of the information obtained in this analysis allowed a risk score to be allotted to each patient. Grouping of patients by the prediction scores is found in this series to be more closely related to survival than is clinical staging of the same patients.**

THE large number and variety of papers published on carcinoma of the breast, the treatment, prognosis and associated factors, are an index of the complexity of the problem and indeed of the study of any tumour. Because this is a relatively common lesion, studies of many aspects have been possible. As a rule one aspect in particular has been examined and a relationship to prognosis or aetiology has been revealed. However, the importance of a particular factor and its independence of, or dependence upon, other factors can only be assessed if many factors in one group of patients are assessed and analysed.

Factors which have been shown to influence the prognosis in carcinoma of the breast in the female include the following: age (McKenzie, 1955), menopausal status (MacMahon, List and Eisenberg, 1968), size of tumour (McWhirter, 1957), delay (Registrar General, 1967), clinical stage (Paterson, Tod and Russell, 1939), axillary node involvement (Myers, Axtell and Zelen, 1966), size of axillary nodes (Fisher, Slack and Bross, 1969), internal mammary node involvement (Handley and Thackray, 1954), malignancy grading of tumour (Bloom, 1950), host response (Hamlin, 1968), hormone balance (Hayward, Bulbrook and Greenwood, 1961), serum cholesterol levels (Juret, Aubert and de Kaouël, 1967), treatment (Bloom, Richardson and Harries, 1962).

It is clear from this list that many of these factors are interdependent. Age and menopausal status are related and both are probably related to hormone balance and perhaps to serum cholesterol levels. The size of the tumour, the delay in seeking advice, the clinical stage, and the node involvement are almost

* Present address: Department of Medical Information Science, University of Southampton.

certainly reflections of a balance between the malignancy grading of the tumour and the host defence response.

However, information about a tumour and the host is, in the last analysis, only of value if this information can be of use to guide the clinician in the diagnosis and treatment of the individual patient. In carcinoma of the breast, it is the treatment of the patient which is currently under examination and discussion. The efficacy of any particular treatment can only be assessed if the influence of pre-treatment clinical and pathological factors are known. Various forms of treatment can be compared if all the pretreatment factors known to influence the prognosis are measured and their influence is allowed for.

The factors mentioned above do not provide a complete list; there are others which may be relevant, and future work will no doubt add to the list (as well as excluding some of those mentioned). With this number of variates to be considered in an examination of the determinants of survival, the problem is complex; a planned study requires large numbers of patients from whom a wide range of information must be collected. The wealth of data from such a study will be rather confusing and far removed from classic scientific experiment where one alters one factor at a time in order to identify the action of cause and effect. Myers, Axtell and Zelen (1966) have drawn attention to this problem, and suggest that a special analysis is required to study simultaneously the effect of several variates. The technique that they used only enabled them to use data from four variates at one time; this falls far short of the requirements in breast cancer. Material is now reported for 21 of the relevant independent variates; this has been analysed in a way which attempts to disentangle the independent effects that each factor exerts on survival.

### MATERIAL

In a previous paper, Hamlin (1968) presented evidence supporting the hypothesis that the prognosis of a patient with carcinoma of the breast is dependent on the host reaction to the tumour as well as the grade of malignancy of the tumour. The material was derived from 272 patients treated at the Royal Marsden Hospital by radical mastectomy between 1935 and 1945, who either died from their carcinoma, or were alive 15 years or more after initial treatment. In the original analysis a large number of factors were assessed, but in the final analysis and in the present multivariate analysis, the following 21 factors were used.

### DEFINITION OF FACTORS

*Clinical stage.*—The original hospital records were examined by a single observer (M.D.S.); from an evaluation of the clinical facts each case was staged according to the TNM system. Clinical size was present for only 137 of the 258 patients; the staging has therefore had to be done without reliance on tumour size.

*Duration of symptoms.*—The duration of symptoms in months preceding initial hospital treatment.

*Age.*—The age of the patient recorded at the time of initial hospital treatment.

*Menopausal status.*—Menopausal status recorded at time of initial hospital treatment. For women for whom this information was not present in the notes, those aged 45–49 were grouped with menopausal women; those outside this age range were grouped with pre- and post-menopausal women.

*Marital status.*—Marital status recorded at time of initial hospital treatment.

*Site.*—The site of the primary recorded in the clinical notes before treatment (the following six sites were used: upper inner, lower inner, upper outer, lower outer, central and axillary).

*Laterality.*—Origin of primary in left or right breast.

*Pathological size.*—The size in centimetres as recorded at the pathological examination of the primary. For a number of specimens no measurement had been made but it was possible in most cases to assess the size of the tumour from the histological preparations available for study.

*Axillary node involvement.*—Axillary nodes were recorded as positive or negative, without comment as to which or how many nodes contained metastases or whether a single node contained just one peripheral metastasis or was completely replaced.

*Malignancy grading.*—As in Hamlin (1968), the malignancy grading used was a modification of that described by Bloom (1950) with division of patients into well differentiated tumours (approximately equivalent to Bloom Grade I) and the rest (equivalent to Bloom Grades II and III).

*Shape of the edge of the tumour.*—Carcinomas of the breast vary considerably in the shape of the infiltrating edge of the tumour, but essentially can be divided into three groups: a " round " edge, a serrated edge and a third group where no true edge is present, the tumour cells infiltrating as cords of single cells over a large ill-defined area.

*Stroma of the tumour.*—Here the word stroma applies to the supporting tissue of the tumour which is either loose and vascular with little collagen present in it (as in the " medullary " carcinoma), or is densely collagenous (as in the " scirrhous " carcinoma), or is a mixture of these two. In the original analysis a number of groups were recognised but were finally amalgamated to form two groups giving the predominant picture of either " loose fibrovascular " or " collagenous ".

*Host defence reaction factors.*—(See Hamlin (1968) for details.)

(i) Lymphocytic and plasmacytic infiltration around the tumour.
(ii) Lymphocytic and plasmacytic infiltration within the tumour.
(iii) The number, size and activity of the germinal centres in the axillary nodes.
(iv) The immunoblast content of the cortex of the axillary nodes.
(v) The plasma cell content of the axillary nodes.
(vi) The sum of these five.

*Sinus histiocytosis of axillary nodes*, as defined by Black and Speer (1958). This was scored as 0, +, ++, and +++ in the original analysis (Hamlin, 1968) and was used in the present analysis in this form.

*Mast cell infiltration of the edge of the tumour.*—This was scored as 0, +, ++, +++ in the original analysis.

*Year of treatment.*—Year in which radical mastectomy was performed.

*Radiotherapy.*—This was recorded as immediate post-operative irradiation given or not given. All cases receiving preoperative radiotherapy were excluded.

### STATISTICAL METHOD

Hamlin (1968) discussed the relationship of host defence response to tumour grading, clinical staging, nodal metastasis, age and menopausal status. The main emphasis was on the relationship between host defence response and survival. Some sub-groups of the data were analysed in relation to survival; for instance survival was provided by class of host defence reaction within a particular malignancy grade. In a complex multifactorial situation the examination of subgroups of the data is one method of trying to identify evidence of a direct, independent effect of a particular factor on survival. This approach has a major disadvantage as it involves splitting the data into smaller and smaller numbers; interpretation thus becomes difficult due to the diminished weight that can be placed upon such small numbers. An alternative statistical analysis of this material is now presented.

There is a variety of statistical techniques suitable for investigation of the class of problem posed here where the effect of a range of prognostic factors on a particular outcome is to be examined. Initially one can examine the direct association between each of the factors and outcome, and also the extent to which the "independent" factors are inter-related. A multivariate analysis can then be applied which has three advantages. First it enables one to examine the entire set of data and quantify in a relatively simple form the independent contribution that each of the factors makes to outcome. The computer programme used for this, of course, does not appreciate the natural history of breast cancer and arbitrarily divides overlapping associations in order to identify the independent contributions, but it certainly provides one method of disentangling the multifactorial determinants of survival. This may aid the understanding of the natural history of the disease. Secondly, where a number of factors have been recorded which involve time-consuming investigation, multiple regression analysis may demonstrate the part that each of these factors plays in predicting survival; this may indicate how increased weight may be placed upon certain of the items, thus dispensing with the need in future studies to assess the remainder. Thirdly, the analysis enables one to derive scores that can be used to classify the patients into low and high risk. By virtue of its power to assess a wide range of factors, and weight these in a consistent way, it may be possible to refine the accuracy of prediction of outcome. With suitable safeguards, this statistical prediction may be used in the consideration of alternative treatment strategies for individual patients.

Linear multiple regression is applicable if one can assume that each of the factors exerts a direct linear effect on the particular outcome that is being studied, and that for any particular value of the predictor factor the values for the outcome are normally distributed about a mean. The effect on survival of the factors listed above has been examined. Survival was recorded in years and this could be examined in relation to clinical stage scored as 1, 2 or 3; survival decreases from stage to stage, and it can be assumed that the association is sufficiently close to a linear one for the application of the multiple regression model. The size of the tumour, the duration before initial treatment, the age of the patient and the year of treatment were all quantitative items; the scores used were derived from grouping the data. All the histological factors used have been scored on scales such that each increment is presumed to be related to a change in survival. The location of the primary lesion was allotted to one of six sites; the literature does not provide clear cut information about the relationship of each of these sites to survival. The material for the 258 patients was therefore examined and the sites ranked in order of increasing mean survival. This rank, which is shown in Table I, was used as the score for site in the next phase of the analysis.

A study of the literature relating to the influence of the menopause suggests a better prognosis for those women who are in the immediate premenopausal interval and possibly for those who are menopausal. Information about the prognosis of the young and the old relative to the menopausal group is not well documented. In the absence of clear direction, two groups were formed; patients stated to be menopausal with those in the immediate premenopausal interval and the young premenopausal women with the post menopausal.

Five other items were included where there were only two alternative answers, *i.e.* whether or not axillary nodes were involved on histological examination, whether or not radiotherapy was given as part of initial treatment, whether the lesion was in the right or left breast, whether the patient was married or not, and whether the stroma was densely collagenous or loosely fibrovascular. Each of these items has been given an arbitrary code to identify which of the two subgroups the patient falls into; the analysis used does not appear to be unduly affected by the use of such dichotomies in the data. Complete information was available for 159 patients; for an additional 99 subjects information was missing for menopausal status. These were allocated a score according to age (see above).

TABLE I.—*Mean Survival for 258 Patients by Site of Breast Lesion*

| Site | Number of patients | Mean survival in years | Rank* |
|---|---|---|---|
| Central | 39 | 5·8 | 1 |
| Upper inner | 40 | 7·4 | 2 |
| Lower outer | 22 | 8·0 | 3 |
| Lower inner | 22 | 8·7 | 4 |
| Upper outer | 129 | 10·7 | 5 |
| Axillary | 6 | 11·7 | 6 |

* This rank was used in the multivariate analysis as the appropriate score for lesions in each of the six sites.

The remaining 14 patients were excluded because one set of information was not available. Thus the analysis of 21 factors was performed upon data from 258 patients treated by radical mastectomy.

### RESULTS

The computer programme produced a correlation matrix in which the correlation between every possible pair of factors is provided; this gave an output with a very large number of pairs of comparisons. This was extremely useful in looking at the direct association between any of the factors and survival, and the inter-relationship between each of the pairs of factors. Table II provides the correlation coefficients for each of the factors against survival, *i.e.* a measure of the overall association between each factor and survival.† The factors have been listed in the table in order of decreasing size of the correlation. The complete correlation matrix has not been printed, but is available on request to the authors.

The multiple regression analysis of the data provides estimates of the independent contribution of each of the factors to survival. These are given in Table III in a modified form, being converted to the percentage of the total

† The correlation coefficient can vary between +1 and −1; in this example + indicates that survival increased with increasing score, whilst — indicates that survival decreased with increasing score. A perfect linear association between any factor and survival would be indicated by a coefficient of one (1). The absence of any association whatsoever would result in a coefficient of zero (0).

TABLE II.—*Correlation Coefficient Between Each of 22 Prognostic Factors and Survival for 258 Patients With Breast Cancer*

| | | | |
|---|---|---|---|
| Clinical stage | −0·41 | Immunoblasts in axillary nodes | 0·12 |
| Axillary node metastases | 0·40 | Infiltration around the tumour | 0·11 |
| Size of primary | −0·35 | Laterality | 0·09 |
| Stromal reaction | −0·32 | Duration of symptoms | 0·09 |
| Radiotherapy given | 0·28 | Plasma cell content in axillary nodes | 0·08 |
| Site of primary | 0·23 | Mast cell infiltration in tumour | 0·08 |
| Malignancy grading | −0·21 | Germinal centres in axillary nodes | 0·07 |
| Year of treatment | 0·21 | Age | −0·07 |
| Host defence response* | 0·16 | Sinus histiocytosis in axillary nodes | 0·06 |
| Infiltration within the tumour | 0·15 | Edge of tumour | 0·04 |
| Menopausal status | 0·14 | Marital status | −0·02 |

If correlation coefficient $>0\cdot12$ or $<-0\cdot12$ then $P<0\cdot05$
$>0\cdot16$ or $<-0\cdot16$ then $P<0\cdot01$

* Host defence response derived from combined score of five items; lymphocytic and plasmacytic infiltration around, and within the tumour; the number, size and activity of the germinal centres in the axillary nodes; the immunoblast content of the axillary nodes; and the plasma cell content in the axillary nodes.

TABLE III.—*Percentage of Total Variance in Survival Independently Explained by Each of 22 Prognostic Factors for 258 Patients With Breast Cancer*

| | % | | % |
|---|---|---|---|
| Axillary node metastases | 7·6 | Menopausal status | 1·1 |
| Clinical stage | 7·0 | Immunoblasts in axillary nodes | 0·6 |
| Size of primary | 6·4 | Sinus histiocytosis in axillary nodes | 0·4 |
| Radiotherapy given | 3·6 | Duration of symptoms | 0·4 |
| Stromal reaction | 3·5 | Mast cell infiltration in tumour | 0·3 |
| Malignancy grading | 3·4 | Laterability | 0·2 |
| Year treatment | 3·4 | Plasma cell content in axillary nodes | 0·2 |
| Site of primary | 3·0 | Age | 0·1 |
| Host defence response† | 2·9 | Edge of tumour | 0·0 |
| Infiltration around the tumour | 1·4 | Marital status | 0·0 |
| Infiltration within the tumour | 1·3 | Germinal centres in axillary nodes | 0·0 |

If variance explained $>1\cdot5\%$ $P<0\cdot05$
$>2\cdot6\%$ $P<0\cdot01$

† See footnote to Table II.

TABLE IV.—*Comparison of Observed Survival With (a) Clinical Stage, (b) "Prediction" Score Based on all Prognostic Factors, for 151 Patients Sampled by Years of Survival From 258 Women With Breast Carcinoma*

| | Observed survival | | |
|---|---|---|---|
| | 1 year | 5–10 years | 15 years+ |
| (a) Clinical stage | | | |
| I | 1 | 7 | 36 |
| II | 3 | 10 | 32 |
| III | 22 | 17 | 23 |
| (b) Prediction score‡ | | | |
| Low risk | 0 | 5 | 59 |
| Intermediate | 6 | 19 | 27 |
| High risk | 20 | 10 | 5 |

‡This score was calculated for each patient by an application of weights, derived from the multivariate analysis, to the actual patient data for each factor. The distribution of all the scores was then arbitrarily divided into three groups, giving the above association between prediction score and survival.

variance in survival explained by each factor.‡ Again the factors have been listed in the table in descending order of magnitude.

Using data derived from the multivariate analysis it is possible to derive " prediction " scores for each patient in the study, such that a high score indicates likelihood of long survival and a low score indicates short survival. These scores are based on mathematical manipulation of the actual data recorded for each patient in this study. The prediction scores used for Table IV are based on data for all the factors, including histological study of the axillary nodes, and presents a comparison between clinical staging and the prediction score as prognostic instruments.

Table V shows the relationship between prediction score, derived from the limited range of factors available when the operation performed has not included removal of the axillary nodes, and survival.

TABLE V.—*Comparison of Observed Survival and (a) Clinical Stage, (b) " Prediction " Score, Based on Restricted List of Prognostic Factors,* for* 151 *Patients Sampled by Years of Survival From* 258 *Women With Breast Carcinoma*

| | Observed survival | | |
|---|---|---|---|
| | 1 year | 5–10 years | 15 years+ |
| (a) Clinical stage | | | |
| I | 1 | 7 | 36 |
| II | 3 | 10 | 32 |
| III | 22 | 17 | 23 |
| (b) Prediction score† | | | |
| Low risk | 0 | 7 | 57 |
| Intermediate | 9 | 16 | 27 |
| High risk | 17 | 11 | 7 |

* Patient's age, marital and menopausal status, duration of lesion; clinical stage and site; size of primary, malignancy grading, lymphocytic and plasmacytic infiltration in and around the tumour, stromal reaction. These items are available from history, clinical examination and histology of primary lesion.

† See footnote to Table IV.

### DISCUSSION OF RESULTS

Examination of data can be followed by presentation of the results in tables, graphs and histograms. In a complex multifactorial situation, such as the study of prognosis in breast cancer, presentation of results in this way may over simplify the situation and lead to misinterpretation of the data. This paper reports a more complex mathematical analysis of a set of data; this, however, brings with it problems in the presentation of the results. An attempt has been made to present these in as simple a way as is possible in order that the reader may himself judge the interpretation of the data.

Twenty-one separate prognostic factors were available for study in relation to the observed survival for 258 patients with breast cancer; a twenty-second factor

‡ If the data for each patient correctly ranked each patient in order of actual survival, the sum of the variance " explained " would be 100% ; if the data bore no relationship to survival, 0% would be explained. In the data presented here, 44% of the variance in survival is explained on summing the contributions of the 21 independent factors. The unexplained portion is due to a combination of errors in the measurement of the factors included, other factors known to be relevant, but not included in the study, unknown factors and chance. In biological problems such as this, it is unusual to explain as high a percentage as in this study.

was a score for host defence response obtained by addition of the scores for five of the factors (infiltration round tumour, infiltration in tumour, germinal centres, immunoblasts, and plasma cell infiltration of the axillary nodes) (Table II). Eight of the factors had a correlation coefficient with survival that was significant at the 1% level (*i.e.* $P < 0{\cdot}01$). The one factor significant at this level that was surprising was " year of treatment "; all the patients had had initial surgery between 1935 and 1945 and this finding suggests that the results of treatment improved towards the end of the period.

The multivariate analysis carried out on the data enabled an estimate to be made of the independent contribution that each of the factors made to survival; such an analysis takes into account the degree of overlap between the factors before defining the independent contribution that each factor makes towards survival. Table III lists the factors in order of their independent contribution. The same eight factors head the list and have predictive values significant at the 1% level; a ninth factor, host defence reaction has now entered this group. In addition, the analysis has re-ordered the factors to a minor extent. Axillary node metastases, clinical stage, and pathological size of primary still head the list. There is considerable overlap between these three factors, but removal of this by the analysis leaves each factor with a highly significant independent contribution to survival. There is a positive correlation in Table II against " radiotherapy given "; this indicates that survival was poorer amongst those given radiotherapy as part of the initial treatment. Table III shows a highly significant independent effect; this cannot be explained therefore by differences between those treated with and without therapy for stage, malignancy, axillary node involvement, size of tumour, or other recorded factors. Whether the surgeons had selected poor risk patients for radiotherapy on account of other more subtle and unrecorded reasons cannot be demonstrated.

Two histological factors appear next in the list; the malignancy grading and stromal reaction; these and the combined score for the five factors contributing to host defence response each make an independent contribution to survival, that is significant at the 1% level. The factor " site of primary " is probably artificially high in the list due to the method of allocating scores to the individual sites within the breast.

The importance of the quality of the fibrous stroma present in the tumour is surprising. In an early paper (Hueper, 1932), the dense fibrous stroma present in a scirrhous carcinoma was interpreted as a protective scarring and in that analysis, the presence of this type of stroma did appear to be associated with a marginally better prognosis. This may, of course, have had something to do with the proportion of cases which were of malignancy grade I, since a large proportion of grade I tumours do have a " scirrhous " stroma and of course Grade I tumours have a 50% 15 year survival. In the present study where the analysis is taking cognisance of this fact, and the stroma of the tumours was divided into essentially fibrovascular or essentially collagenous or " scirrhous ", a statistically significant difference in prognosis is found, the better group containing those cases having a fibrovascular stroma. This finding, although noted and discussed by Hamlin (1968) was not included then in the H.D.R. score, since there is no evidence that this is an immunological reaction, although clearly it is a host response.

Approximately equal numbers of clinical Stage III patients in this series died within a year of initial treatment and survived more than 15 years; clinical staging, though high on the list in Table III, is unable to adequately identify a discrete group. Using data for all the 21 individual prognostic factors, a prediction score has been calculated and the patients arbitrarily divided into three categories. The results given in Table IV indicated a closer association between prediction score and survival than between clinical stage and survival. A prediction score was also calculated from a limited set of data, without use of information from pathology of the axillary nodes. The results are presented in Table V; the prediction is again much more closely associated with actual survival than is clinical stage.

### GENERAL DISCUSSION

The importance of clinical stage and axillary node metastases in the prognosis of carcinoma of the breast has been confirmed by this analysis and although clearly related, each does have an independent contribution to make to the assessment of prognosis. It is unfortunate that in the original analysis the proportion of axillary nodes involved was not noted. Cutler *et al.* (1969) observed a positive association between survival and the proportion of nodes involved.

The size of the primary in this work was the size of tumour measured by the pathologist on the cut surface of the tumour and in this analysis a clear relationship to prognosis has been found. Clinical size of tumour is more difficult to assess and there does not appear to be a clear relationship between clinical size and pathological size. An attempt to analyse the factors involved in this discrepancy is at present being made on this and another series of patients.

In this analysis the contribution made by the stromal reaction in the tumour is apparently independent of other histological factors such as the malignancy of the tumour, the shape of the tumour infiltration, and the lymphocytic and plasmacytic infiltration of the stroma. Nevertheless while the histological analysis was being made it was observed that a dense lymphocytic and plasmacytic infiltration was often associated with a loose fibrovascular stroma. Is it possible that even in the absence of a visible cellular infiltration, circulating lymphocytes have easier and more intimate contact with the tumour when the stroma is loose and vascular and that the immunological defences are therefore more efficient in these cases? The variation in the density of the collagenous stroma does not appear to be related to the size of the tumour, since a loose fibrovascular stroma may be seen in large tumours and a dense collagenous stroma in very small tumours.

The reason for the clearly poorer prognosis of patients receiving irradiation in this series will remain a subject of debate. Depression of host immunological defences in the crucial post-operative period, when carcinoma cells released into the blood stream during operation are establishing themselves, is certainly a possibility. What is clear from this analysis is that prognosis in breast carcinoma treated by radical mastectomy is not improved by post-operative irradiation.

Conflicting views have been expressed in the literature on the relationship of site in breast to prognosis (Truscott, 1947; Smithers *et al.*, 1952; Treves and Holleb, 1958) but generally it is accepted that inner quadrant tumours have a poorer prognosis. Certainly in this series, patients with tumours in the upper outer quadrant formed the largest group and also showed a high mean survival.

The degree of differentiation of a tumour was first recognised to be of importance in prognosis by Von Hansemann in 1893 and all grading systems of carcinoma are based upon this histological feature. The findings in this analysis confirm the independent contribution which the malignancy of the tumour makes to prognosis.

The relationship of the histological features interpreted by Hamlin (1968) as an immunological host defence reaction to survival has been confirmed by this analysis. It is clear that the different factors making up the host defence score vary in importance and that the cellular infiltration in and around the tumour in the breast has more influence on prognosis than the histological changes in the axillary nodes (*i.e.* other than metastases). This has been taken into account in designing the second predictive index (see below).

Of the factors which are not significant statistically, it is interesting to observe in Table III that " immunoblasts in the axillary nodes " has a much higher figure than " plasma cell content of axillary nodes " or " germinal centres ". This would be in agreement with the present view that effective host defence in carcinoma is cellular rather than humoral.

The prediction scores, derived from this analysis, show a hopefully close association with prognosis. However Armitage, McPherson and Copes (1969) in discussing advanced breast cancer, point out that a method of prediction is likely to be less effective on subsequent data than it appears to be when applied retrospectively to the data from which it was derived. Obviously the weights calculated from the present series will have to be applied to a fresh set of material in order to test the validity of the predictive instrument. This work is now in progress.

If the predictive instrument is found effective when applied to a second fresh collection of retrospective patient data, it may be possible to use it prospectively on data from individual patients immediately after operation as a guide to further clinical management. The current surgical trend is away from radical surgery and there may therefore be no material from the axilla for histological study. The second prediction score has been calculated on such clinical and pathological data as would be available after excision biopsy or simple mastectomy.

### REFERENCES

ARMITAGE, P., McPHERSON, C. K. AND COPES, J. C.—(1969) *J. chron. Dis.*, **22**, 343.
BLACK, M. M. AND SPEER, F. D.—(1958) *Surgery, Gynec. Obstet.*, **106**, 163.
BLOOM, H. J. G.—(1950) *Br. J. Cancer*, **4**, 259.
BLOOM, H. J. G., RICHARDSON, W. W. AND HARRIES, E. J.—(1962) *Br. med. J.*, ii, 213.
CUTLER, S. J., BLACK, M. M., MARK, T., HARVEI, S. AND FREEMAN, C.—(1969) *Cancer, N.Y.*, **24**, 653.
FISHER, B., SLACK, N. H. AND BROSS, I. D. J.—(1969) *Cancer, N.Y.*, **24**, 1071.
HAMLIN, I. M. E.—(1968) *Br. J. Cancer*, **22**, 383.
HANDLEY, R. S. AND THACKRAY, A. C.—(1954) *Br. med. J.*, i, 61.
HAYWARD, J. L., BULBROOK, R. D. AND GREENWOOD, F. C.—(1961) *Mem. Soc. Endocr.*, **10**, 144.
HUEPER, W. C.—(1932) *Ann. Surg.*, **95**, 321.
JURET, P., AUBERT, C. AND DE KAOUËL, C. R. B.—(1967) *Path. Biol., Paris*, **15**, 515.
McKENZIE, A.—(1955) *Lancet*, ii, 1129.
MACMAHON, B., LIST, N. D. AND EISENBERG, H.—(1968) in ' Prognostic Factors in Breast Cancer ', edited by A. P. M. Forrest and P. B. Kunkler. London (Livingstone), p. 56.
McWHIRTER, R.—(1957) *J. Fac. Radiol.*, **8**, 223.
MYERS, M. H., AXTELL, L. M. AND ZELEN, M.—(1966) *J. chron. Dis.*, **19**, 923.

PATERSON, R., TOD, M. C. AND RUSSELL, M. H. (1939) 'Five Year Statistical Report on the Results of Radium Therapy for the Years 1932 and 1933'. Stockport (Rowland and Berry).
REGISTRAR GENERAL—(1967) Statistical Review for England and Wales, 1961. Supplement on Cancer. London (H.M.S.O.).
SMITHERS, D. W., RIGBY-JONES, P., GALTON, D. A. G. AND PAYNE, P. M. (1952) *Br. J. Radiol.*, Supplement No. 4.
TREVES, N. AND HOLLEB, A. I.—(1958) *Surgery Gynec. Obstet.*, **107**, 271.
TRUSCOTT, B. McN.—(1947) *Br. J. Cancer*, **1**, 129.